# Effect of acupuncture and massage on adolescent idiopathic scoliosis and pain severity

**DOI:** 10.3389/fmed.2025.1613800

**Published:** 2025-07-21

**Authors:** Kun Jia, Yan Yang

**Affiliations:** ^1^Department of Rehabilitation, Beijing Children's Hospital Affiliated to Capital Medical University, Beijing, China; ^2^Traditional Chinese Medicine Department, Beijing Children's Hospital Affiliated to Capital Medical University, Beijing, China

**Keywords:** adolescent idiopathic scoliosis, acupuncture, massage, pain, Cobb Angle, quality of life

## Abstract

**Objective:**

To explore the impact of acupuncture and massage on adolescent idiopathic scoliosis (AIS) and its influence on pain degree.

**Methods:**

One hundred and sixteen adolescent patients with idiopathic scoliosis treated from January 2021 to December 2023 were chosen to be the study objects, followed by dividing into control group and observation group. The control group received brace correction combined posture training. The observation group received traditional Chinese medicine (TCM) acupuncture and bone-setting massage treatment. The Cobb Angle, degree of pain, therapeutic effect, quality of life, incidence of adverse reactions and spinal function were compared in 2 groups.

**Results:**

After therapy, the Cobb Angle value and VAS score were declined in 2 groups, and those in the observation group presented lower when comparing with the control group (*P* < 0.01). In contrast to the control group, the total effective rate in the observation group was better (*P* < 0.05), and the SRS-22 score in the observation group was higher (*P* < 0.05). After therapy, the ODI score was declined and JOA score was elevated in 2 groups, and the improvements of ODI and JOA scores in the observation group were more significant when comparing with the control group (*P* < 0.01).

**Conclusion:**

Acupuncture and massage can promote the clinical treatment effect, reduce the degree of pain, promote Cobb Angle recovery, promote the quality of life and improve the spinal function of AIS.

## Introduction

Adolescent idiopathic scoliosis (AIS) is a common musculoskeletal disorder that affects growth and development in adolescents, with a Cobb Angle >10° serving as the primary diagnostic criterion (1). AIS often presents with vertebral rotation as the initial symptom, followed by physical abnormalities such as uneven shoulder height, thoracic asymmetry, leg length discrepancies, rib humps, pelvic tilt, and reduced height (2). In severe cases, AIS may lead to cardiopulmonary dysfunction, neurological paralysis, and elevated surgical risk (35). Furthermore, the physical deformities associated with AIS can contribute to psychological distress, including low self-esteem and mental health challenges (3). Leg length discrepancy in AIS patients results from pelvic tilt secondary to spinal curvature, while height reduction stems from vertebral rotation and compression of intervertebral spaces (36). Nowadays, AIS has become another significant health concern after adolescents' health faces obesity and myopia (4). According to the survey, 0.47–5.20% of the world's adolescents suffer from AIS, and the number of primary and secondary school students in China has exceeded 3 million, and the number of about 300,000 is increasing year by year (5).

At present, the clinical treatment of AIS mostly uses orthoses or conservative management such as chiropractic, physiotherapy exercise, and axial traction (37). Traditional Chinese medicine (TCM) believes that AIS belongs to the category of “spinal deformity”, and its pathogenesis is closely related to the imbalance of muscles and bones caused by internal and external pathogenic factors invading the body (6). Acupuncture and massage are traditional Chinese medicine therapy, with safety, affordable, effective and other advantages (7). Acupuncture can effectively promote the circulation of qi and blood in the back area, improve the microcirculation of nerve roots, and achieve the balance of muscle strength on both sides of the spine (8). Massage can make the paraspinal muscles and soft tissues fully relaxed, and then look for the scoliosis segment, with the adjustment of the manipulation to correct the vertebral misalignment, so as to restore the normal physiological curvature of the spine (9).

In our study, we explored the effect of acupuncture combined with massage on AIS and its influence on pain degree.

## Data and methods

### Participants

A total of 116 adolescent patients with idiopathic scoliosis treated between January 2021 and December 2023 were included in the study. Patients were randomly assigned to either the control group or the observation group, with 58 cases in each group. The control group included 30 males and 28 females. The average age was (12.56 ± 1.28) years, ranging from 10 to 16 years. Type of scoliosis: 23 cases in thoracolumbar segment, 18 cases in thoracic segment, 17 cases in lower back segment. There were 29 males and 19 females in the observation group. The average age was (12.52 ± 1.25) years, ranging from 9 to 17 years. Type of scoliosis: thoracolumbar segment in 24 cases, thoracic segment in 19 cases, low back segment in 15 cases. There was no significant difference in general data between 2 groups (*P* > 0.05). Inclusion criteria: (1) Consistent with AIS diagnostic criteria; (2) The age range was 9–17 years old; (3) Conformance with conservative treatment and Cobb Angle <45°; (4) The legal guardian signed the relevant consent. Exclusion criteria: (1) Patients with congenital spinal malformation; (2) Severe spinal cord injury with cardiopulmonary dysfunction; (3) Participants who had received other physical and exercise therapy interventions within 1 month before participating in this study; (4) People with mental illness or cognitive disorder (Table 1).

**Table 1:** Baseline Cobb Angle distribution by scoliosis type in control and observation groups.

**Groups**	**Curve type (Cobb Angle range)**	**Mean Cobb Angle**
Control	Thoracolumbar (15°-35°): 23 cases Thoracic (12°-30°): 18 cases Lumbar (10°-28°): 17 cases	24.5°± 6.2°
Observation	Thoracolumbar (16°-34°): 24 cases Thoracic (13°-32°): 19 cases Lumbar (11°-29°): 15 cases	25.1°± 5.8°

### Methods

The control group received brace correction combined posture training. Appropriate chest, waist, and sacral braces were selected according to convex vertex, and were worn for no <21 h every day. The change of height was closely paid attention to, and the brace was replaced or adjusted as appropriate. Nurses guided the patient to perform the chest expansion exercise with feet equally equal to shoulder width, assisted the patient to pull the horizontal bar, and asked the patient to pay attention to relax the body, and then stand against the wall, so that the calf, hip, heel and shoulder joints were close to the wall, and the arms were naturally drooping, repeat the above training, 3–5 times/d, 10 min/times. At the same time, nurses asked patients to pay attention to sitting and standing posture in life.

The observation group received TCM acupuncture and bone-setting massage treatment.

#### Bone-setting massage

The patient was placed in a prone position, and the muscles around the curved spine were stretched for 20 min. Then, the doctor pressed the convex side of the scoliosis with one palm and the opposite shoulder with the other hand, and gave the reaction force along the spine from top to bottom for 5 min. The bone-setting massage was performed twice a week. During the massage, the doctor should pay attention to gentle manipulation.

#### Acupuncture

The doctor selected YaoShu point, HuanTiao point, ZhiYang point, Sanyinjiao point, Ququan point, Jizhong point, Mingmen point, and Shenzhu point. When acupuncture was performed, 6–8 lumbosacral acupuncture points and 2–4 leg acupuncture points were selected, radian needle expansion method was applied, and moderate stimulation was given. Acupuncture was performed according to WHO Standard Acupuncture Point Locations (38), with needle retention for 30 min.

The intervention was performed for 3 months in both groups.

### Observation indicators

(1) The Cobb Angle of the patient was measured on the full-length anteroposterior X-ray film of the spine. The upper and lower vertebrae were located, respectively. Two straight lines were drawn along the upper edge of the upper vertebrae and the lower edge of the lower vertebrae, and then the Angle between the vertical lines of the two lines was measured, which was called Cobb Angle.
(2) Visual analog method (VAS) was implemented for evaluating the pain degree of patients (10). The total score was 10 points. The lower the score was, the less the pain was.
(3) The therapeutic effect criteria are formulated following the Guidelines for the Diagnosis and Treatment of Common Diseases of Traditional Chinese Chiropractic and Chinese Chiropractic. Cured: Radiographic examination showed that the scoliosis deformity disappeared, the signs and symptoms disappeared, and the Cobb Angle was <5°. Effective: Radiographs showed less scoliosis than before, less symptoms, improved function, Cobb Angle decreased ≥5°. Ineffective: Radiographs showed no significant changes in scoliosis, Cobb Angle decreased <5°, and even the scoliosis Angle became larger than before. Total effective rate = (cured cases + effective cases)/Total cases × 100%.
(4) At the end of treatment, the Scoliosis Research Society-22 Patient Questionnaire (SRS-22) was implemented for evaluating the quality of life of patients (11). The full score of the scale was 100, and the higher the score, the better the quality of life.
(5) The incidence of adverse reactions including dizziness, nausea, vomiting, tissue damage and fracture in 2 groups were analyzed.
(6) Oswestry Disability Index (ODI) together with Japanese Orthopedic Association (JOA) was implemented for assessing the spinal function of patients (12, 13). The full score of ODI scale was 50, and the full score of JOA scale was 29. The lower the ODI score, the better the spinal function. The higher the JOA score, the better the spinal function.

### Statistical analysis

SPSS 24.0 statistical software was adopted for data analysis. Measurement data were expressed as (*x* ± *s*), and *t*-test was adopted for comparison. Count data were expressed as (*n*, %), and χ^2^ test was used for comparison. *P* < 0.05 meant statistical significance.

## Results

### Cobb Angle value in 2 groups

**Figure 1:**
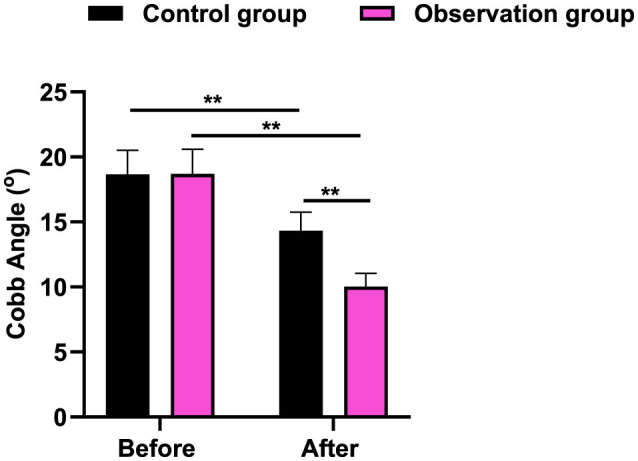
Cobb Angle value in 2 groups. **P < 0.01.

It was revealed in Figure 1 and Table 2 that, the no difference was seen in Cobb Angle value between 2 groups before therapy (*P* > 0.05). After therapy, the Cobb Angle value was declined in 2 groups, and that in the observation group presented lower when comparing with the control group (*P* < 0.01).

**Table 2:** Comparison of primary outcomes between groups.

**Outcome**	**Control group**	**Observation group**	***P*-value**
Cobb Angle (°)	18.2 ± 4.1	12.6 ± 3.8	<0.01
VAS score	3.5 ± 1.2	2.1 ± 0.9	<0.01
SRS-22 score	78.4 ± 6.3	85.7 ± 5.1	0.013

### VAS score in 2 groups

**Figure 2:**
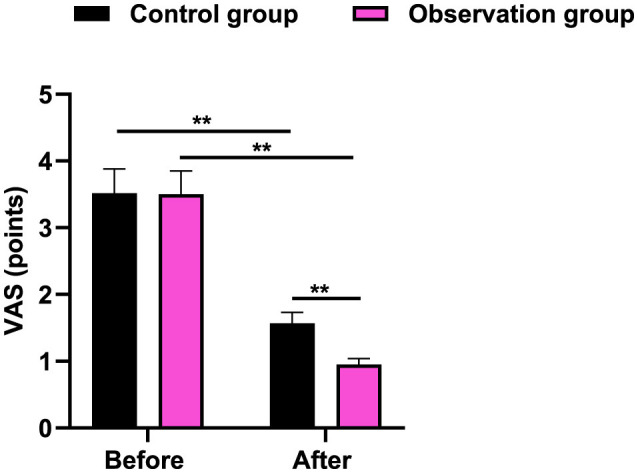
VAS score in 2 groups. **P < 0.01.

It was revealed in Figure 2 that, the no difference was seen in VAS score between 2 groups before therapy (*P* > 0.05). After therapy, the VAS score was declined in 2 groups, and that in the observation group presented lower when comparing with the control group (*P* < 0.01).

### Therapeutic effect in 2 groups

Table 3 displayed that in contrast to the control group, the total effective rate in the observation group was better (*P* < 0.05).

**Table 3:** Therapeutic effect in 2 groups.

**Groups**	***N***	**Cured**	**Effective**	**Ineffective**	**Total effective rate**
Control group	58	15	33	10	48 (82.75%)
Observation group	58	20	36	2	56 (96.55%)
χ^2^					5.949
*P*					0.014

### SRS-22 score in 2 groups

**Figure 3:**
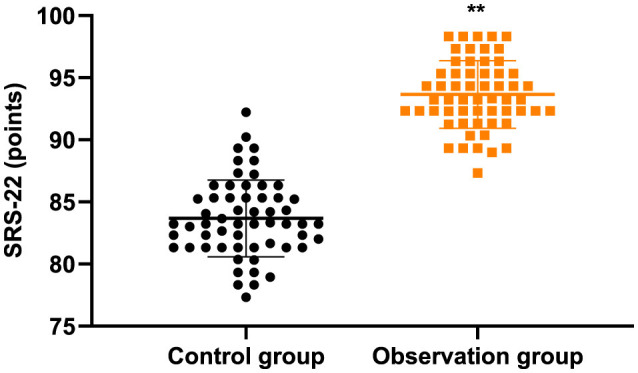
SRS-22 score in 2 groups. **P < 0.01.

Figure 3 and Table 2 displayed that in contrast to the control group, the SRS-22 score in the observation group was higher (*P* < 0.05).

### Incidence of adverse reactions in 2 groups

There were no adverse reactions containing dizziness, nausea, vomiting, tissue injury, and fracture in the 2 groups during treatment.

### Spinal function in 2 groups

**Figure 4:**
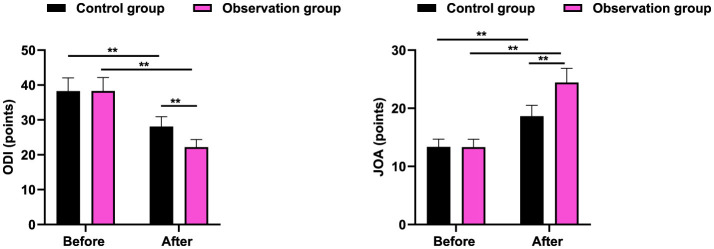
Spinal function in 2 groups. **P < 0.01.

It was revealed in Figure 4 that, the no difference was seen in ODI and JOA scores between 2 groups before therapy (*P* > 0.05). After therapy, the ODI score was declined and JOA score was elevated in 2 groups, and the improvements of ODI and JOA scores in the observation group were more significant when comparing with the control group (*P* < 0.01).

## Discussion

Scoliosis is a common clinical condition that can cause contracture of the soft tissues around the spine, resulting in tensile tension, further aggravating the severity of scoliosis (14). Due to the rapid physical development of adolescents, vertebral bones will be further enlarged, if not timely improvement of scoliosis symptoms can seriously affect the normal development of the spine, affecting study, life, and work (15).

TCM believes that scoliosis belongs to the “turtle back” category, and its pathogenesis is considered to be related to the invasion of bone by external and internal pathogens and the loss of muscles and bones, and the imbalance of tendons and bones is an important pathological change (16). At present, there are many clinical treatment methods for AIS, including bone-setting massage and acupuncture, which have certain therapeutic effects (17).

Bone-setting massage can correct vertebral subluxation, facilitate qi and blood flow by stimulating the Du meridian, and promote the recovery of spinal function (18). The release of soft tissue on both sides of the spine can promote scoliosis recovery and facilitate stability after bone-setting. Massage on the concave side of the contracture tissue can make it fully relax and relieve the contracture state. Massage from top to bottom along the side bend can help further relax the contracture tissue, and then bone setting can obtain better results (19). The vertebral displacement can be corrected by boning, the normal anatomical structure of the joint can be restored, and the scoliosis deformity can be effectively corrected. Bone-setting massage is a non-invasive method, basically will not produce side effects and adverse reactions, will not cause secondary damage, easy to be accepted (20).

Acupuncture is a common external treatment of TCM. Based on the meridian theory of TCM, acupuncture can improve the muscle and ligament strength corresponding to the meridian and its distribution tissues by stimulating specific acupoints (21). Currently, acupuncture therapy has been widely applied in the rehabilitation treatment of patients with spinal injuries (22). In this study, the YaoShu point of acupuncture points belongs to the governor meridian, which can dispel cold and dehumidification, and the combination with HuanTiao point can be used to treat the hip pain symptoms caused by waist diseases such as AIS (23). Zhiyang point also belongs to the governor meridian, which can be used to treat strong back pain (24). Sanyinjiao point is the spleen channel, can promote blood and qi (25). Ququan point belongs to the liver channel of foot Jueyin, which can be used for activating channels and clearing dampness and heat (26). The Jizhong point and Mingmen point belong to the governor meridian, which can be used to treat the severe pain of the lumbar spine (27). The Shenzhu point is also a governor meridian, which can be used to calm god and relieve convulsion (28). Acupuncture stimulation of the above points can effectively improve the function of muscles and ligaments in the area covered by the corresponding meridians, and achieve the effect of relaxing muscles and facilitating blood circulation, removing blood stasis and stopping circulation (29). Relevant studies have shown that acupuncture can increase local blood flow by stimulating corresponding acupuncture points, promote nutrient delivery and waste metabolism by improving blood circulation around the spine, and regulate nerve function by stimulating nerve endings (30). In addition to improving the shape of the spine, it can also effectively promote the recovery of spinal function in patients (31).

In our study, the results indicated that after therapy, the Cobb Angle value and VAS score were declined in 2 groups, and those in the observation group presented lower when comparing with the control group. Meanwhile, in contrast to the control group, the total effective rate in the observation group was better. All these results suggested that acupuncture and massage could promote the clinical treatment effect, reduce the degree of pain, and also promote Cobb Angle recovery. Consistently, Wei et al. have pointed that TCM combined therapy can prevent the progression of scoliosis (8). These findings align with prior reviews indicating that adolescent idiopathic scoliosis may benefit from conservative and manual therapy approaches (33, 34).

Besides, our study indicated that in contrast to the control group, the SRS-22 score in the observation group was higher, and after therapy, the ODI score was declined and JOA score was elevated in 2 groups, and the improvements of ODI and JOA scores in the observation group were more significant when comparing with the control group. All these results indicated that acupuncture and massage could promote the quality of life and improve the spinal function of AIS. Likewise, Huang et al. have suggested that massage and acupuncture has a significantly superior effect on reducing chronic spinal pain and improving spinal function (32).

Nevertheless, there were still some limitations for this study. The 3-month follow-up period may not capture long-term effects. Moreover, blinding of practitioners was impractical due to the nature of manual therapies. Future studies should include radiographic assessments beyond Cobb Angle, such as rotational measurements via CT.

In conclusion, Acupuncture combined with massage significantly reduced Cobb Angles (mean reduction: 5.6° vs. 2.3° in controls), suggesting its potential as a complementary approach for mild-to-moderate AIS. Acupuncture and massage are effective in reducing pain severity, improving Cobb Angle recovery, and enhancing quality of life and spinal function in adolescents with AIS.

## Data Availability

The datasets presented in this study can be found in online repositories. The names of the repository/repositories and accession number(s) can be found in the article/supplementary material.
